# Effect of chitosan coating containing *Nepeta pogonosperma* extract on shelf life of chicken fillets during chilled storage

**DOI:** 10.1002/fsn3.2429

**Published:** 2021-07-05

**Authors:** Fatemeh Afshar Mehrabi, Akram Sharifi, Maryam Ahvazi

**Affiliations:** ^1^ Department of Food Science and Technology Faculty of Industrial and Mechanical Engineering Qazvin Branch Islamic Azad University Qazvin Iran; ^2^ Medicinal Plants Research Center Institute of Medicinal Plants ACECR Karaj Iran

**Keywords:** chemical changes, chicken fillet, chitosan, microbial quality, *Nepeta pogonosperma* extract, shelf life

## Abstract

Chicken meat is highly susceptible to microbial and chemical spoilage due to its high moisture and protein content. The use of edible coatings contains herbal extracts with antioxidant and antibacterial properties that help to extend the shelf life of meat products. In this study, the effect of chitosan coating (2%) and *Nepeta pogonosperma* extract (NPe) (0.2% and 0.6%) and their combination on chemical properties (pH, peroxide value (PV), thiobarbituric acid index (TBARS), total volatile basic nitrogen (TVB‐N)) and microbial (aerobic mesophilic and psychrotrophic microorganisms, lactic acid bacteria, *Enterobacteriaceae* and *Pseudomonas* sp.) of chicken fillets were studied over a 12‐day refrigerated storage period compared to the control sample. The results of NPe DPPH radical scavenging activity (DRSA) showed that IC_50_ and total phenolic contents values were 94.65 μg/ml and 113.53 mg GAE/g extract, respectively. Statistical results showed that the rate of increase in pH, PV, TBARS, and TVB‐N of all coated treatments were lower than control. Microbial analysis results showed a decrease in the growth of different bacteria in chitosan‐treated combined with NPe compared to the control sample during chilled storage. Chicken fillets coated with chitosan and 0.6% NPe displayed a longer shelf life compared to other samples.

## Graphical abstract

This study aimed to investigate the effects of chitosan coating and Nepeta pogonosperma extract and their combination on chemical and microbial properties of chicken fillets were studied over a 12‐day refrigerated storage period compared to the control sample.

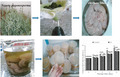

## 1 INTRODUCTION

Chicken is the cheapest and most accessible source of meat. Unlike beef or pork, there are no religious or cultural restrictions on chicken consumption that have made chicken meat a high consumption in the world (Ozunlu et al., 2018). Digestible proteins (low in collagen): unsaturated fats, B‐group vitamins (thiamin, vitamin B_6,_ and pantothenic acid) and minerals such as iron, copper, and zinc make chicken meat a valuable food. The high protein and moisture content of chicken meat provides a good medium for the growth of microorganisms, so chicken meat has a much shorter shelf life, while causing significant economic damage to producers due to the potential presence of pathogens it also threatens consumers’ health. Reports have shown that chicken meat consumption was the first cause of food poisoning prevalence in the United States between 1998 and 2012 (Konuk Takma & Korel, 2019; Raeisi et al., 2016; Rouger et al., 2017). In addition to microbial degradation, aerobic conditions in meat cause proteins and lipid oxidation. Lipid oxidation can negatively affect the sensory properties of the product such as color, texture, taste, odor, crunchiness, and also the nutritional quality of meat (Gomez & Lorenzo, 2012; Karre et al., 2013). Natural or synthetic antioxidants can reduce or inhibit the oxidation process in meat and meat products, thus enhancing the quality and shelf life of meat products. Antioxidants such as butylated hydroxyanisole (BHA), butylated hydroxytoluene (BHT), propyl gallate (PG), and tertiary butylhydroquinone (TBHQ) are synthetic antioxidants that are widely used in meat products (Shah et al., 2014). But today the use of these chemicals has been limited due to their deleterious effects on DNA and their toxicity. Recently, the tendency to use plant extracts has increased as natural additives in foods to protect them against oxidation and prevent the growth and proliferation of microorganisms (Sayari et al., 2015).

One of the *Nepeta* species was scientifically identified as *Nepeta pogonosperma* (NP) as a new species in 1984 (Jamzad & Assadi, 1984). Local people in the Alamut area (Qazvin province, Iran) use flowering aerial of the plant to flavor dairy products and preserve meat (Ahvazi et al., 2007). The extract yield of the flowering aerial parts of the NP is 1.7% and the major compounds in its essential oil included 1,8‐cineole, 4aα, 7α, 7aα‐nepetalactone, β‐Pinene, terpinene‐4‐ol, α‐terpineol, linalool, 4aα, 7β, 7aα‐nepetalactone, delta‐terpineol, geranyl acetate, α‐Pinene. NP generally has antioxidant activity, and phenolic compounds are much higher than other species of *Nepeta* (Khalighi‐Sigaroodi et al., 2013).

If the essential oil and herbal extracts are just added to food, they may alter their organoleptic properties due to their high concentration. Edible coatings can be considered as a suitable carrier for these compounds (Fernandez‐Pan et al., 2014). Chitosan is the second natural polymer, cellulose being the first. One of the interesting properties of chitin and chitosan in food packaging is their antimicrobial and antifungal properties, which enhance the immunity and longevity of food products (Dhall, 2013).

However, to our knowledge, to date, the combination of chitosan and *Nepeta pogonosperma* extract has never been produced and tested in fresh chicken fillets. In this study, the effects of chitosan coating and *Nepeta pogonosperma* extract (NPe) on chemical and microbial properties of chicken fillets were studied during chilled storage.

## 2 MATERIALS AND METHODS

### 2.1 Materials

In this research, *Nepeta pogonosperma* (NP) was collected in August 2018 from Piche Bon village of Alamut region in Qazvin province (Iran) and was authenticated by a botanist. A voucher specimen of the plant has been deposited in the central herbarium (*mpih.ir*) with code no. 527(MPIH). Aerial parts of NP were dried in shade at room temperature (25°C). After complete drying, the aerial parts were milled (DELMONTI‐DL125) and then kept in dark glass bottles. All chemicals in analytical grades were purchased from Merck Company. The bacterial strain cultures were obtained from the *Iranian Research Organization for Science and Technology* (IROST).

### 2.2 Preparation of extract

20 g of the plant powder was mixed with 200 ml of methanol, and the extraction process was done by an ultrasonic bath (DT 255 H, Bandelin Co. Germany) for 2 hr (Khalighi‐Sigaroodi et al., 2013). The NPe was filtered using filter paper Whatman 40 and concentrated at a low temperature (<50°C) using a vacuum rotary evaporator (BUCHI‐ water bath B‐480, Flawil, Switzerland). The concentrated extract was stored in air‐tight dark glass bottles and kept refrigerated (4°C) for further treatments after solvent separation (Sharifi et al., 2015).

### 2.3 Determination of DPPH radical scavenging activity

DPPH radical scavenging activity (DRSA) of NPe was evaluated by the DPPH test (2, 2‐diphenyl‐1‐picrylhydrazyl). First, 0.5 ml of DPPH solution was mixed with 4.5 ml of methanol. Then, 0.1 ml of the extract was added at various concentrations (25–100 μg/ml) and mixed for 1 min. The mixture was incubated at room temperature for 30 min; its absorbance at 517 nm was read using a spectrometer (PerkinElmer‐LAMBDA35). The DPPH scavenging activity of each sample will be calculated by Equation (1).

(1) $$\text{DRSA}\;\left(\%\right)=\;\left(A_{\text{blank}}‐\;A_{\text{sample}}/\;A_{\text{blank}}\right)\;\times \;100$$

where *A*_blank_ and *A*_sample_ control and extract absorbance at 517 nm, respectively.

The EC_50_ value was calculated as the concentration at which the DPPH radical scavenging activity was 50% (Zhang et al., 2016).

### 2.4 Determination of total phenolic contents

Total phenolic contents (TPC) was measured by Folin–Ciocalteu method. In this method, 20 µl of the extract was mixed with 1.16 ml distilled water and 100 µl of Folin–Ciocalteu reagent. 1 to 8 min later, 300 µl of sodium carbonate solution (20%) was added and stored at room temperature for 30 min. The absorbance was calculated at 765 nm by a spectrophotometer. The results were expressed in terms of mg Gallic Acid Equivalents per g of extract (Slinkard & Singleton, 1977).

### 2.5 Microbiological analysis

#### 2.5.1 Disc diffusion method

Antibacterial activity of NPe was measured with agar disc diffusion assay. Microorganisms used were *E. coli* (ATCC 25922), *Pseudomonas aeruginosa* (ATCC9027), and *Salmonella enterica* (ATCC10708). Nutrient agar medium was prepared, autoclaved, and transferred aseptically to sterilize Petri plates. 100 µl of bacterial suspension (10^8^ CFU/ml) was spread on plates, and then, circular disc (6.4 mm) was impregnated with 20 µl of NPe. The discs were placed over plates of Muller Hinton agar seeded with each bacteria, and the inoculum was adjusted to 0.5 Mc Farland turbidometry. The plates were incubated at 37°C for 24 hr. Chloramphenicol (30 µg/disc) was applied as a positive control to determine the sensitivity of one strain in each microbial species tested. The zones of inhibition around each of the discs were calculated by measuring the diameter in mm as a measure of the antimicrobial activity after incubation time (Prasannabalaji et al., 2012).

#### 2.5.2 Minimum inhibitory concentration (MIC) and minimum bactericidal concentration (MBC) of NPe

The MIC is the lowest concentration of NPe that will inhibit the visible growth of a microorganism after overnight incubation. The MBC is the lowest concentration of NPe required to kill a particular bacterium, it can be determined from MIC tests by subculturing to agar plates that do not contain the test agent. In this study, MIC and MBC were measured according to the method of Prasannabalaji et al. (2012).

### 2.6 Preparation of coating solution containing NPe and treatment of chicken meat

Chitosan powder with medium molecular weight was dissolved in 1% acetic acid to produce a 2% solution. After filtration, 0.75% glycerol was added as a plasticizer and was stirred at room temperature on a hotplate/magnetic stirrer for one hour. Based on MIC and MBC results, the 0.2% and 0.6% of NPe mixed with 2% Tween 80 was added to the chitosan solution. The solution was stirred at room temperature for 30 min (Bazargani‐Gilani et al., 2015).

Skinless and boneless chicken fillets (each slice weight 120 g) were obtained from local distributors in Qazvin, Iran. The samples were placed in a sealed cooler with a layer of ice between the samples and transported to the laboratory of Qazvin Islamic Azad University.

Fillets were divided into six groups, including control sample (chicken fillets dipped in sterile distilled water), chicken fillets dipped in 2% chitosan (Ch) solution, chicken fillets dipped in 2% chitosan solution containing 0.2% NPe, chicken fillet dipped in 2% chitosan solution containing 0.6% NPe, chicken fillet dipped in 0.2% NPe, and chicken fillet dipped in 0.6% of the NPe. Fillets were dipped in coating solutions for 1 min and then removed for 2 min and again dipped in coating solutions for 1 min. The excess solution was drained off immediately after dipping. Finally, all samples were stored in refrigeration condition (4 ± 1°C), and chemical and bacterial tests were performed on storage days 0, 3, 6, 9, and 12 (Jonaidi Jafari et al., 2018).

### 2.7 Determination of pH

The pH value was recorded using a pH meter (Crison GLP 22, EEC). 10 g of the meat sample was mixed with 50 ml of distilled water and homogenized for 1 min then the pH was read (Banerjee et al., 2012).

### 2.8 Determination of peroxide index (PV)

The sample (20 g) was mixed with 100 ml chloroform–methanol (2:1 V/V) in a glass tube and vortexed for 1 min. The chloroform phase was used for the solvent evaporate and fat extraction for peroxide measurement. Chloroform‐acetic acid mixture was added to the fat in a ratio of 2:3. Next, 0.5 ml of a saturated solution of potassium iodide was added and kept in the dark for 5 min and after adding 75 ml of distilled water was titrated with sodium thiosulphate using 0.5 ml of dissolved starch adhesive as the indicator. PV was expressed as milliequivalents peroxide per kg of fat (AOAC, 2005; Jonaidi Jafari et al., 2018).

### 2.9 Determination of thiobarbituric acid reactive substances (TBARS)

Thiobarbituric acid reactive substances formed due to lipid peroxidation during storage were determined using the method of Ozunlu et al. (2018) 5 g of chicken fillets was blended with 50 ml of trichloroacetic acid (20%). Then, 5 ml of filtered solution was mixed with 5 ml of 0.02 M thiobarbituric acid solution and heated in a boiling water bath at 80℃ for 35 min and cooled and the absorbance was measured at 532 nm The amount of TBARS was expressed as mg malonaldehyde per kg of the sample (Ozunlu et al., 2018).

#### 2.9.1 Determination of total volatile basic nitrogen (TVB‐N)

After the addition of magnesium oxide powder to the minced chicken fillet, TVB‐N content was determined by distillation. The distillate was collected in a flask containing a 3% (w/v) aqueous solution of boric acid and a mixed indicator produced by dissolving 0.1 g of methyl red and 0.05 g methylene blue to 100 ml ethanol. The boric acid solution turned green when the distilled TVB‐N made it alkaline. The boric acid solution was titrated with a 0.01 mol/L chloric acid solution until it turned pink (Cao et al., 2013).

$$\text{TVB}‐N\;\left(\text{mg}/100\;g\right)\;=\;\left(V\;\times \;C\;\times \;14\;\times \;100\right)/10.$$

where V and C volume and concentration of HCl, respectively.

#### 2.9.2 Color measurement

The color of chicken fillets was measured by using a Hunter laboratory Instrument (TES)(135A‐Taiwan). L* (lightness) represents the brightness on a scale of (dark) to 100 (white), a* (redness) scale ranges from negative values for green to positive values for red and b* (yellowness) scale ranges from negative values for blue to positive values for yellow (Yuan et al., 2016).

#### 2.9.3 Microbiological analysis

Chicken fillet (10 g) was mixed with 90 ml of buffered water in a sterile plastic bag and homogenized in the stomacher (Seward Ltd) for 60 s. Appropriate dilutions were prepared in tubes containing 0.1% buffered water and cultured by the pour plate method. Culture media of Aerobic mesophilic bacteria, *Enterobacteriaceae,* psychrotrophic bacteria, Lactic acid bacteria, *Pseudomonas* sp. were plate count agar, VRBG, plate count agar, MRS, *Pseudomonas* agar base, respectively (Fernández‐Pan et al., 2014).

#### 2.9.4 Statistical analysis

All experiments were conducted in triplicate, and analysis of variance was performed using SPSS software version 21. The least significant difference at *p* < .05 was calculated using the Duncan multiple range test.

## 3 RESULTS AND DISCUSSION

### 3.1 DPPH radical scavenging activity (DRSA) and total phenolic contents (TPC) of NPe

The results of DRSA evaluation of NPe different concentrations revealed the antioxidant activity increased by increasing the concentration of NPe. The concentration of 25, 50, 75, and 100 μg/ml had 11.65, 25.41, 39.18, and 52.94% DRSA, respectively. NPe concentration of 100 μg/ml had highest DRSA (52.94%) and lowest IC_50_ value (94.65 μg/ml).

In recent years, there has been a global trend toward the use of the natural substances present in medicinal plants with high antioxidant activity and much research has been done by various researchers to evaluate the antioxidant properties of medicinal plants (Lee et al., 2005). The results of Shahsavari et al. (2008) indicated the IC_50_ of *Zataria multiflora* Boiss extract was 2.22 ± 0.04 mg/ml (Shahsavari et al., 2008). The IC_50_ value of the *Nepeta cataria* methanol extract was 171.98 μg/ml (Adiguzel et al., 2009). According to the results and compared with previous studies, NPe has high antioxidant activity.

The amount of phenolic compounds of NPe was 113.53 mg GAE/g extract. Phenolic compounds are secondary metabolites of plants. These compounds have high antioxidant potential and are effective in removing and preventing free radicals (Wong et al., 2006). Increasing the concentration of phenolic compounds directly increases the radical scavenging activity in plant extracts (Zhang et al., 2009). Sharifi et al. (2019) and Shahidi et al. (2020) reported phenolic compounds of 329.815 mg GAE/100 ml for barberry fruit (*Berberis Vulgaris*) extract and 141.598 mg GAE/100ml for flixweed (*Descurainia sophia*) seeds extract, respectively (Shahidi et al., 2020; Sharifi et al., 2019).

### 3.2 Evaluation of the antimicrobial activity of NPe

#### 3.2.1 Minimum inhibitory concentration (MIC) and minimum bactericidal concentration (MBC) of NPe

NPe showed good antibacterial activity against tested microorganisms. The MIC values of the NPe against the *E. coli*, *S. entrica,* and *P. aeruginosa* were found in the range of 0.78–6.25 mg/ml (Table 1).

**TABLE 1:** Minimum inhibitory concentration (MIC) and minimum bactericidal concentration (MBC) of *Nepeta pogonosperma* extract (NPe)

Bacteria	MIC(mg/ml)	MBC(mg/ml)
*E. Coli*	0.78	1.56
*S. Entertica*	6.25	6.25
*Ps. Aeruginosa*	3.12	6.25

**FIGURE 1:**
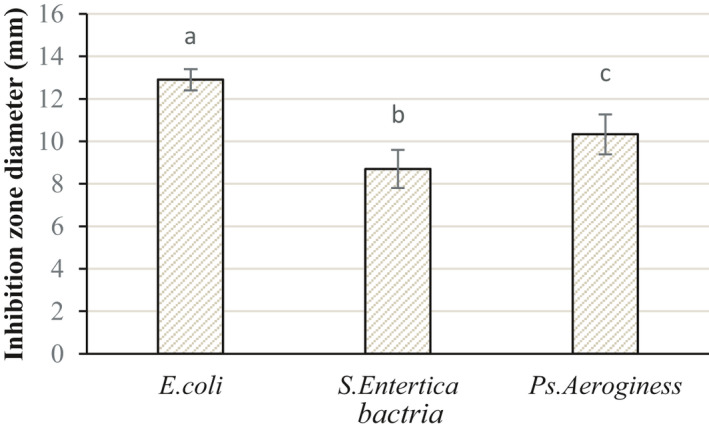
Effect of *Nepeta pogonosperma* extract (NPe) on inhibition zone diameter (mm)

The results obtained from the disc diffusion method indicated that the highest (12.0 ± 9.5 mm) and the lowest (8.0 ± 7.89 mm) zone of inhibition diameters belonged to *E. coli* and *S. entrica*, respectively (Figure 1). There is a significant difference in the zone of inhibition values for the three bacterial strains (*p* < .05).

### 3.3 pH

On day zero of storage, pH values of the treatments varied from 5.57 to 5.54, and no significant differences were observed between the samples at a level of 0.05. Over time, pH values of the samples increased slightly during all days of storage (*p* < .05) (Table 2). On the 12th day of storage, the control and chitosan treatment containing 0.6% of NPe had the highest (6.17 ± 0.005) and the lowest (6.02 ± 0.005) values of pH, respectively. This rising trend is in line with the report of Hassanzadeh et al. (2018) concerning the effect of chitosan coating containing grape seed extract on the shelf life of rainbow trout fillet and that of Kostaki et al. (2009) regarding the shelf life of *Dicentrarchus labrax* fillet, resulting from the production of alkaline compounds such as trimethylamine and ammonia by bacteria (Hassanzadeh et al., 2018; Kostaki et al., 2009). The low pH in chicken meat coated with chitosan and NPe during storage can also be attributed to their microorganisms growth inhibitory potential and protease enzymes (Fan et al., 2009; Pabast et al., 2018) and acidic pH of chitosan (Hassanzadeh et al., 2017).

**TABLE 2:** Effect of chitosan (Ch) and *Nepeta pogonosperma* extract (NPe) coating on the pH of chicken fillets during storage at 4°C

Treatment	Storage time(day)				
	0	3	6	9	12
Control	5.57 ± 0.005^eA^	5.91 ± 0.01^dA^	6.01 ± 0.005^cA^	6.11 ± 0.005^bA^	6.17 ± 0.005^aA^
Ch	5.54 ± 0.015^eA^	5.75 ± 0.005^dB^	5.86 ± 0.01^cC^	5.96 ± 0.005^bD^	6.06 ± 0.02^aD^
Ch +NPe (0.2%)	5.56 ± 0.015^eA^	5.62 ± 0.015^dC^	5.85 ± 0.015^cC^	5.95 ± 0.005^bD^	6.05 ± 0.03^aD^
Ch +NPe (0.6%)	5.57 ± 0.01^eA^	5.58 ± 0.015^dD^	5.81 ± 0.01^cD^	5.94 ± 0.005^bD^	6.02 ± 0.005^aE^
NPe (0.2%)	5.56 ± 0.015^eA^	5.77 ± 0.01^dB^	5.91 ± 0.005^cB^	6.08 ± 0.005^bB^	6.12 ± 0.01^aB^
NPe (0.6%)	5.57 ± 0.005^eA^	5.74 ± 0.01^dB^	5.87 ± 0.005^cC^	6.06 ± 0.01^bC^	6.11 ± 0.005^aC^

Note

Different uppercase letters in the same column and lowercase letters in the same row indicate a significant difference (*p* < .05).

### 3.4 Peroxide value (PV)

Lipid oxidation in meat leads to off‐flavor and degraded quality. Peroxide is formed in the early stages of oxidation as a result of oxygen binding to double bonds of unsaturated fatty acids. Therefore, initial lipid oxidation can be assessed by measuring PV (Cao et al., 2013). According to Table 3, there was no significant difference between the treatments on day zero of storage. With increasing storage time, however, PVs increased in all samples, particularly in the control sample (*p* < .05), with the highest PV (7.11 ± 0.04 meq/kg) in the control sample on the 12th day of storage. On the 12th day, peroxide levels were significantly lower in all coated treatments than the control treatment, with the chitosan coating having 0.6% of NPe containing the lowest level (4.47 ± 0.09 meq/kg).

**TABLE 3:** Effect of chitosan (Ch) and *Nepeta pogonosperma* extract (NPe) coating on the peroxide value (PV) (meq/kg) of chicken fillets during storage at 4°C

Treatment	Storage time(day)				
	0	3	6	9	12
Control	2.03 ± 0.096^eA^	3.64 ± 0.048^dA^	4.06 ± 0.048^cA^	5.44 ± 0.048^bA^	7.11 ± 0.048^aA^
Ch	2.00 ± 0.083^eA^	3.14 ± 0.048^dB^	3.86 ± 0.048^cB^	5.08 ± 0.083^bB^	6.19 ± 0.096^aB^
Ch + NPe (0.2%)	2.03 ± 0.048^eA^	2.92 ± 0.083^dD^	3.72 ± 0.048^cC^	4.50 ± 0.083^bC^	5.56 ± 0.048^aC^
Ch + NPe (0.6%)	2.03 ± 0.096^eA^	2.78 ± 0.127^dE^	3.56 ± 0.048^cD^	4.11 ± 0.096^bD^	4.47 ± 0.096^aE^
NPe (0.2%)	2.06 ± 0.048^eA^	3.14 ± 0.048^dB^	3.89 ± 0.048^CB^	5.19 ± 0.048^bB^	6.17 ± 0.083^aB^
NPe (0.6%)	2.06 ± 0.048^eA^	3.04 ± 0.041^dC^	3.81 ± 0.048^cC^	4.58 ± 0.083^bC^	5.22 ± 0.048^aD^

Note

Different uppercase letters in the same column and lowercase letters in the same row indicate a significant difference (*p* < .05).

During the storage period, there was no significant difference between the treatment with chitosan coating and that containing 0.2% NPe. Results show the advantage of pure NPe coatings and it can be concluded that pure NP coating could reduce the production of hydroperoxides and decelerate the oxidation process, the same as 2% chitosan solution whose antioxidant activity was proven in various studies (Darmadji & Izumimoto, 1994; Inanli et al., 2020; Ojagh et al., 2010; Zhang et al., 2020). Plant extracts, including NPe, cease oxidative chain reactions by donating hydrogen to free radicals, thereby exerting their antioxidant effects (Abdou et al., 2018). In line with the results of this study, Ojagh et al. (2010) reported that PVs increased in all treatments of coated rainbow trout meat, but this increase was lower in chitosan and chitosan plus cinnamon essential oil treatments during the storage period (Ojagh et al., 2010). Bazargani‐Gilani et al. (2015) also presented evidence that PV increased in coated chicken fillets until the end of the 10th storage day and then decreased until the end of day 20 due to degradation of hydroperoxides (Bazargani‐Gilani et al., 2015).

### 3.5 Thiobarbituric acid reactive substances (TBARS)

**FIGURE 2:**
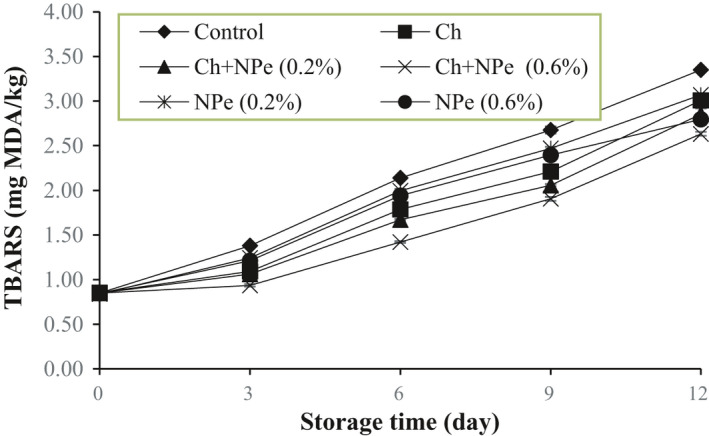
Effect of chitosan (Ch) and *Nepeta pogonosperma* extract (NPe) coating on the thiobarbituric acid reactive substances (TBARS) (mg MDA/kg) of chicken fillets during storage at 4°C

Changes in the TBARS index during the storage period generally showed significantly increased levels in all treatments of this study until the end of the storage period. No significant differences were observed between the treatments on day zero (*p* > .05), and TBARS level (0.85 ± 0.006 mg MDA/kg) in the control sample reached 3.35 ± 0.01 mg MDA/kg after 12 days of refrigerated storage, with the highest increase compared to the other treatments. The lowest changes in TBARS were observed in chitosan‐coated samples containing 0.2% and 0.6% of NPe, where a TBARS level of 0.85 mg MDA/kg on the first day reached 2.85 ± 0.06 and 2.63 ± 0.02 MDA/kg, respectively, after 12 days of storage (Figure 2). The chitosan‐treated combined with NPe showed better performance in the reduction of lipid oxidation than single use. Additionally, changes in the treatments during the storage days suggest that the antioxidant activity of NPe increased with rising concentration, but the increase was not statistically significant on the third day of storage. In general, the antioxidant activity of such herbal extracts as NP can be related to their secondary compounds, such as phenolic compounds (Jridi et al., 2018). By stabilization of hydroperoxides, phenolic compounds prevent their oxidation and further degradation, and the formation of such compounds as malondialdehyde (Hernández‐Hernández et al., 2009). A report from Mahdavi et al. (2018) also indicated a decrease in the rate of TBARS changes in chicken burgers treated with chitosan and anise essential oil (2 and 1.5%) film (Mahdavi et al., 2018). Qin et al. (2013) also detected that a combination of chitosan coating and tea polyphenols could greatly reduce the oxidation process of pork. (Qin et al., 2013).

### 3.6 Total volatile basic nitrogen (TVB‐N)

**FIGURE 3:**
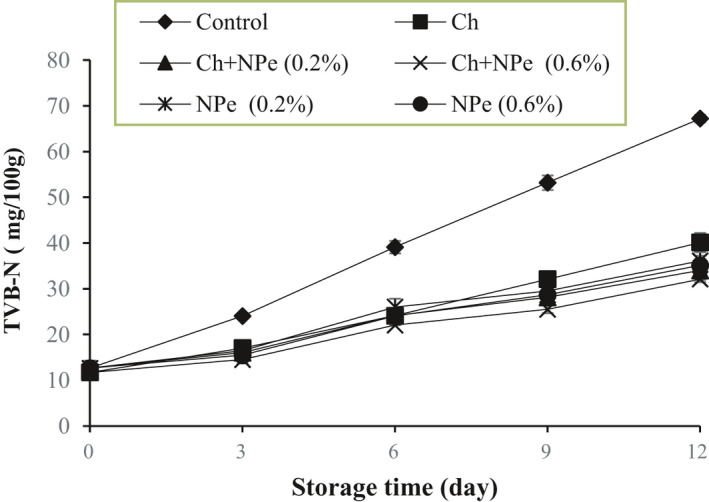
Effect of chitosan (Ch) and *Nepeta pogonosperma* extract (NPe) coating on the total volatile basic nitrogen (TVB‐N) (mg/100 g) of chicken fillets during storage at 4˚C

TVB‐N as one of the indicators of fresh meat detection includes a wide range of volatile compounds such as ammonia, methylamine, dimethylamine, trimethylamine, and other similar compounds produced during the storage of meat in cold conditions due to microbial activity (Anderson, 2008; Rodríguez et al., 2008). According to Figure 3, TVB‐N levels increased significantly with time (*p* < .05). On the 12th day, the highest (67.23 ± 0.08 mg/100 g) and the lowest (32.12 ± 0.7 mg/100 g) amounts of TVB‐N were recorded, respectively, in the control sample and the chitosan treatment contained 0.6% of NPe. A maximum permissible amount of TVB‐N as 28 mg/100 g was announced by the Veterinary Organization of Iran. Accordingly, the control sample with a TVB‐N amount of 39.33 ± 1.4 mg/100 g could not be consumed on the 6th day of storage whereas all the coated treatments were within the allowable limit in terms of TVB‐N levels until the end of the 6th day. On the 9th day, however, TVB‐N levels in all treatments were outside the standard permissible range, and the chitosan‐coated treatment containing 0.6% of NPe with a TVB‐N amount of 25.5 ± 0.8 mg/100 g was only consumable on this day.

The reductions of TVB‐N changes in samples with chitosan coating, NPe, and their combination can be attributed to the antimicrobial properties of chitosan and NPe (Aziz & Karboune, 2018; Goy et al., 2016; Kong et al., 2010), as well as the combined performance and synergistic effect of chitosan coating and the extract (Yuan et al., 2016). Consistent with our findings, Mojaddar Langroodi et al. (2018) investigated the effects of a chitosan coating containing thyme essential oil, sumac extract, and MAP packaging on meat shelf life and reported an increasing trend of TVB‐N during 20 days of storage. They announced that the amount of TVB‐N in samples reached >14 mg/100 g with an increasing total number of bacteria to 10^7^ log_10_ CFU/g. The researchers also reported that chitosan‐coated treatment containing 4% sumac extract and 1% thyme essential oil with the highest antimicrobial properties was the most effective treatment against changes in TVB‐N levels (Mojaddar Langroodi et al., 2018). A very recent research Rezaeifar et al. (2020) also indicates a decrease in TVB‐N changes in rainbow trout fillets coated with chitosan containing *Lemon verbena* essential oil and extract packaged in vacuum conditions. This phenomenon has been attributed to the ability of phenolic compounds to inhibit the growth of microorganisms, thereby preventing the oxidation of lipids and degradation of proteins (Rezaeifar et al., 2020).

### 3.7 Brightness factor (L* value)

**FIGURE 4:**
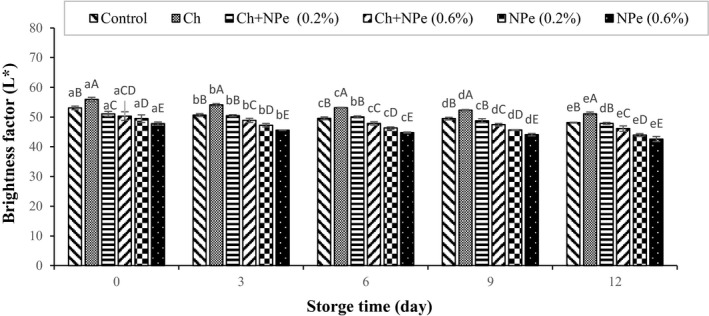
Effect of chitosan (Ch) and *Nepeta pogonosperma* extract (NPe) coating on the brightness factor (L* value) of chicken fillets during storage at 4°C (Different letters of each bar indicate significant difference between the storage time within same analysis group (lowercase) and differences between treatment groups within same analysis day (uppercase) at *p* < .05)

The results of color changes in chicken fillets during the storage period are shown in Figure 4. Nonsignificant changes in a* and b* indices were not investigated here. An increase in the storage time was associated with decreased L* levels in all samples, which reached their minimum value at the end of the storage period. The statistical results further revealed that the highest and lowest L* values belonged to samples coated with pure chitosan and 0.6% NPe, respectively, in the whole storage period.

Hernández‐Hernández et al. (2009) examined the effects of thyme and rosemary on the color of pork samples and observed decreased L* values with increasing TBARS index during storage, with the treatment containing rosemary extract showing the lowest level of L* during the study (Hernández‐Hernández et al., 2009). The addition of grape seed extract and pine bark was also demonstrated to reduce L* levels in cooked beef compared to control treatment in the same storage periods (Ahn et al., 2007), which is similar to our observations. Contrary to the present results, Petrou et al. (2012) noticed elevated L* values in the chicken meat treated with chitosan and thyme (oregano) essential oil during the storage period. They found the lowest L* index in the control treatment in the Map packaging, which was significantly higher in treatments containing thyme, chitosan essential oil, and their combination in the MAP packaging on the 12th day (Petrou et al., 2012). Furthermore, the addition of kinnow rind extract was reported to increase the L* index while adding pomegranate rind powder extract reduced L* levels in pieces of cooked goat meat (Devatkal et al., 2010).

Color is generally one of the most important indicators of meat freshness from the customer's viewpoint (Konuk Takma & Korel, 2019). Since the L* index indicates the darkness and lightness of meat color, its changes can be attributed to the oxidation of lipids and proteins, and its effect on meat pigments(Carvalho et al., 2017; Hernández‐Hernández et al., 2009).

### 3.8 Microbial analysis

**FIGURE 5:**
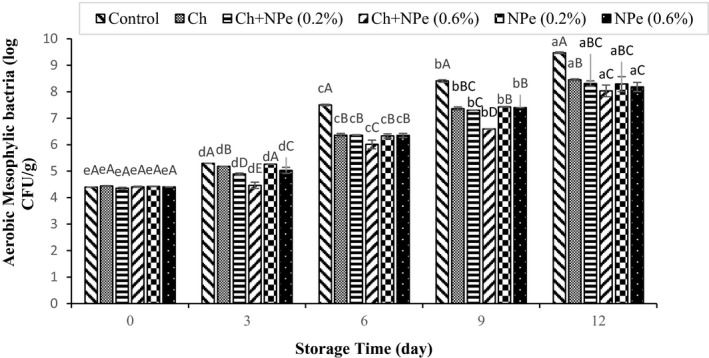
Effect of chitosan (Ch) and *Nepeta pogonosperma* extract (NPe) coating on the total aerobic mesophilic bacteria counts of chicken fillets during storage at 4°C

**FIGURE 6:**
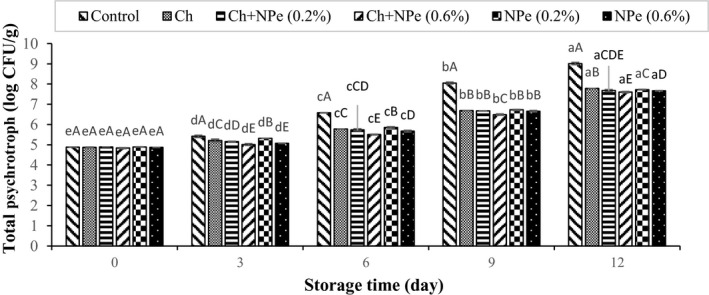
Effect of chitosan (Ch) and *Nepeta pogonosperma* extract (NPe) coating on the psychrotrophic bacteria counts of chicken fillets during storage at 4°C

**FIGURE 7:**
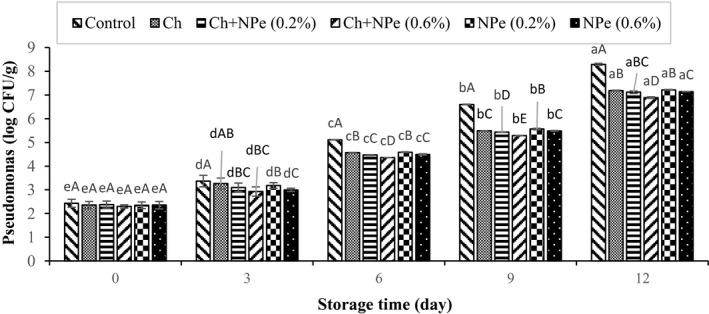
Effect of chitosan (Ch) and *Nepeta pogonosperma* extract (NPe) coating on the *Pseudomonas* bacteria counts of chicken fillets during storage at 4°C

**FIGURE 8:**
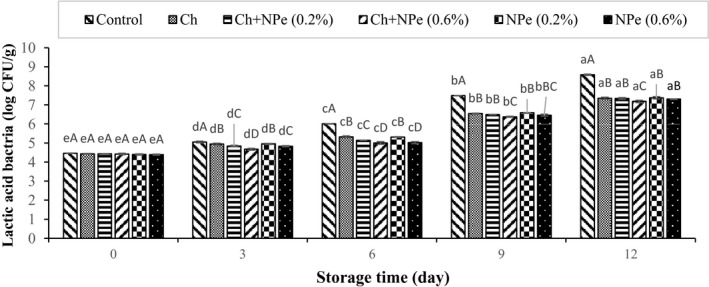
Effect of chitosan (Ch) and *Nepeta pogonosperma* extract (NPe) coating on the lactic acid bacteria counts of chicken fillets during storage at 4°C

**FIGURE 9:**
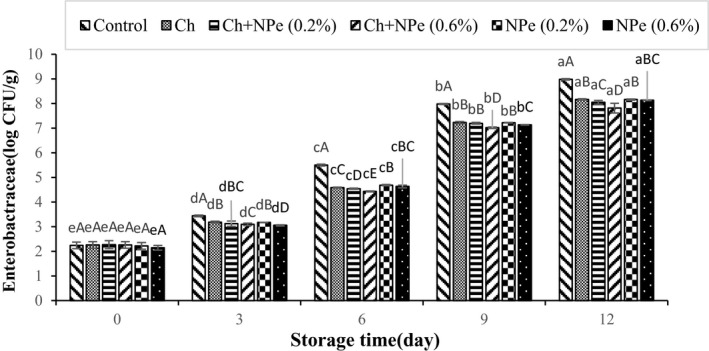
Effect of chitosan (Ch) and *Nepeta pogonosperma* extract (NPe) coating on the *Enterobacteriaceae* bacteria counts of chicken fillets during storage at 4°C

Microbial analysis of samples during storage at 4°C indicated an increasing trend in the populations of aerobic mesophilic, psychrotrophic, *Pseudomonas*, lactic acid, and *Enterobacteriaceae* bacteria (Figures 5, 6, 7, 8, 9). The control treatment and the chitosan treatment containing 0.6% of NPe, respectively, contained the highest and lowest microbial populations during the storage period, except on day zero. A comparison of 0.6% NPe with pure chitosan coating treatments also showed no significant difference in the former treatment on some days of the storage period. When the total number of bacteria in the meat exceeds 7 logarithmic cycles, the meat begins to spoil and changes occur in its organoleptic properties (Eldaly et al., 2018). According to Figure 5, the number of mesophiles in the control treatment reached 7.50 Log CFU/g on the 6th day of storage, while the microbial load of coated treatments was still less than seven logarithmic cycles on the same day when the highest effect of declined microbial growth was also observed in the chitosan treatment containing 0.6% NPe. In this treatment, the number of mesophiles (6.01 ± 0.16 Log CFU/g) shows that the chitosan coating containing 0.6% of NPe was able to reduce the microbial load of fillets by approx. 1.5 logarithmic cycles. On the 9th day of storage, the number of mesophiles in all samples was above the allowable limit (seven logarithmic cycles), except in chitosan treatment containing 0.6% of the extract, meaning that this treatment was able to maintain its microbial quality until the end of the 9th day of storage. In a study concerning the effect of chitosan coating at different concentrations (1, 1.5, and 2%) on the microbial characteristics of chicken fillets during 15 days of storage at refrigerator temperature, the total number of aerobic bacteria reached 6.87 Log CFU/g in the control treatment on the 3rd day of storage, while that of coated samples was 5.99–6.97 Log CFU/g at the end of the 12th day of storage (Eldaly et al., 2018). The growth inhibition of aerobic microorganisms by chitosan was attributed to the formation of chitosan film around the cell membrane and the prevention of oxygen entry into the cells of microorganisms. There are generally several hypotheses about the antimicrobial effects of chitosan, apparently due to the interaction between positively charged chitosan molecules and negative charge of bacterial cell membrane, leading to leakage of proteins, bacterial cell constituents, and eventually death (Yuan, Lv, et al., 2016). Maghami et al. (2019) also examined the effect of chitosan nanoparticles combined with fennel essential oil on fish meat shelf life and concluded that coating significantly reduced the growth of mesophiles, psychrotrophs, lactic acid bacteria, and *Pseudomonas* compared to Map packaging and control treatment (Maghami et al., 2019).

Psychrotrophic bacteria are mainly responsible for the spoilage of meat products at refrigerated temperatures (Bazargani‐Gilani et al., 2015). As shown in Figure 6, the number of psychrotrophs also increased significantly in all samples with increasing storage time (*p* < .05). A comparison of microbial loads of different samples with the allowable limit (seven logarithmic units) showed that the control sample maintained its proper microbial quality until day 6, while psychrotroph counts in the coated samples were within the allowable limit until day 9. Similar to the results of this study, Mehdizadeh and Mojaddar Langroodi. (2019) also reported a decrease in the number of psychrotrophs in chicken specimens with a combination of chitosan coating containing propolis extract and thyme essential oil. They found that the amount of extract and its antimicrobial properties declined after a while if used alone, whereas a combination of chitosan coating and extract led to the extract stability for a certain period. Thus, the extract hydrolyzes the bacterial cell membrane by affecting the surrounding peptidoglycan layer, thereby increasing the antimicrobial effect of chitosan (Mehdizadeh & Mojaddar Langroodi, 2019).

*Pseudomonas* is a gram‐negative, aerobic rod bacterium that grows rapidly in refrigerated conditions (Lu et al., 2016). Due to the strong proteolytic properties of these bacteria, the signs of spoilage appear in fresh meat when the number of these bacteria reaches about 7–8 logarithmic cycles (Mehdizadeh & Mojaddar Langroodi, 2019). In this study, the number of *Pseudomonas* ranged from 2.30 to 2.43 CFU/g in all treatments on day zero, which increased during the refrigerated storage. The highest (8.29 ± 0.04 CFU/g) population of *Pseudomonas* was observed in the control treatment on the last day of storage, and the sample coated with chitosan containing 0.6% NPe was the only treatment in which *Pseudomonas* population was 6.89 ± 0.03 CFU/g by the end of the 12th day and did not exceed seven logarithmic cycles. In addition, the results demonstrated that *Pseudomonas* population decreased with increasing the extract concentration from 0.2 to 0.6 in all test days except day zero, suggesting the elevated antibacterial property of the extract with increasing the concentration (Figure 7). In agreement with this study, Lu et al. (2016) examined the antimicrobial properties of eucalyptus essential oil in vitro and reported a significant decrease in *Pseudomonas* population compared with control treatment after adding 4% eucalyptus concentration to pork (Lu et al., 2016).

The number of lactic acid bacteria also showed a rising trend during storage at refrigerated temperatures, so that the highest (8.58 ± 0.03 CFU/g) and the lowest (7.18 ± 0.05 CFU/g) populations were present in the control sample and the chitosan treatment containing 0.6% of NPe on the 12th day (Figure 8). These findings are in line with those reported for chicken fillets treated with niacin‐containing sodium alginate and cinnamon and rosemary essential oils (Raeisi et al., 2016).

The population of *Enterobacteriaceae*, as facultative anaerobic bacteria, also showed a significant increase with time (Figure 9). This upward trend was observed with much greater intensity in all treatments on the last days of storage, which corresponds to Cai et al. (2018) who studied the effect of combined chitosan coating and herbal (lemon and thyme) essential oils on the fillet shelf life of grass carp (*Ctenopharyngodon idella*) (Cai et al., 2018). Overall, the results of this study demonstrated the antimicrobial properties of chitosan and NPe. Herbal extracts and essential oils have been reported to disrupt the cytoplasmic membrane activity, proton motive force electron flow, and active transport, leading to coagulation of bacterial cell content and consequently death (Nikmaram et al., 2018). The strongest treatment against microbial changes was chitosan treatment containing 0.6% of NPe, which indicates a more effective synergistic effect of chitosan coating and NPe.

## 4 CONCLUSION

The results showed that coating chicken fillets led to decreased pH, peroxide value, thiobarbituric acid index, total volatile basic nitrogen, and microbial counts (aerobic mesophilic, psychrotrophic, lactic acid, *Pseudomonas*, and *Enterobacteriaceae* bacteria) during the storage period. The chicken fillet sample coated with chitosan containing 0.6% of *Nepeta pogonosperma* extract could better retain its chemical and microbial qualities during storage than the other treatments. Considering the potential antioxidant activity and high levels of phenolic compounds in *Nepeta pogonosperma* extract, it can be used in the pharmaceutical and food industries instead of synthetic antioxidants and other chemical preservatives to delay lipid oxidation and inhibit the growth of microorganisms.

## CONFLICT OF INTEREST

The authors declare that they have no conflict of interest.

## AUTHOR CONTRIBUTIONS

**Fatemeh Afshar Mehrabi:** Formal analysis (equal); Funding acquisition (equal); Investigation (equal); Resources (equal); Software (lead). **Akram Sharifi:** Funding acquisition (supporting); Methodology (equal); Project administration (equal); Software (supporting); Supervision (lead); Validation (equal); Writing‐original draft (equal); Writing‐review & editing (equal). **Maryam Ahvazi:** Methodology (supporting); Validation (supporting); Visualization (supporting).

## ETHICAL APPROVAL

This article does not contain any studies with human participants or animals performed by any of the authors.

## Data Availability

The data that support the findings of this study are available from the corresponding author, upon reasonable request.
